# Statistics of Hospital and Out-Patient Service

**Published:** 1888-10

**Authors:** 


### STATISTICS OF HOSPITAL AND OUT-PATIENT SERVICE.

### *Veterinary Department—University of Pennsylvania.*

September 1st, 1887, to September 1st, 1888.

| | HOSPITAL. | OUT PATIENTS. |
|---|---|---|
| Abortion | 2 | |
| Abscess | 8 | 13 |
| Adenitis | 1 | |
| Agagrophila | 1 | |
| Amputation. | | |
| tail horse | 8 | |
| toe dog | | 1 |
| Amaurosis | | 1 |
| Anæmia | 1 | |
| Anasarca | | 4 |
| Anchylosis | 1 | |
| Arthritis | | 2 |
| Asthma | 1 | |
| Atrophy, muscular | | 1 |
| Bronchitis | 6 | 22 |
| " chronic | 1 | 1 |
| Bruised soles | 2 | 2 |
| Bruised heel | 1 | 1 |
| Burnt sole | | 5 |
| Calculi | | |
| intestinal | 1 | |
| vesicular | 2 | |
| Caries | | |
| superior maxillary | | 1 |
| Castration | 2 | 10 |
| Cataract | 1 | 7 |
| Catarrh, auricular | 8 | 13 |
| Cerebral congestion | 1 | |
| Cerebro spinal meningitis | 2 | |
| Chorea | 7 | 12 |
| Chapped foot pad | | 1 |
| Collection of sinuses | 3 | 9 |
| " " guttural pouches | 1 | |
| Conjunctivitis | | 2 |
| Constipation | 2 | 10 |
| Contracted heel | 7 | 25 |
| Contraction of Flexor Tendon | 2 | |
| Contusions | 4 | |
| Convulsions | 1 | 3 |
| Corns | 4 | 14 |
| " supurating | 2 | 4 |
| Curb | | 1 |
| Cyst, serous | 1 | 6 |
| " mucous | 4 | 2 |
| Deafness | 1 | |
| Diarrhœa | 2 | 2 |
| Dilatation of carpal sheath | | 1 |
| Dislocation of eye | 1 | |
| Distemper | 12 | 27 |
| Dropsy, brain | 1 | |
| " abdominal | 2 | |
| Dystochia | 2 | |
| Ears, cut | 3 | 6 |
| " deformed | 1 | |
| Eczema | 21 | 39 |
| Elephantiasis | 1 | 2 |
| Emboli | 1 | 1 |
| Emphysema | 1 | 5 |
| Enteritis, chronic | 2 | |
| Entorrhagia | 1 | |
| Entropia | 1 | |
| Epilepsy | 1 | |
| Erythema | | 1 |
| Enucleation of eyes | 2 | |
| Examination for soundness | 3 | 50 |
| Exostosis of carpas | 1 | |
| Feet, deformed | | 5 |
| Fever, purpureal | 1 | |
| Fistula. | | |
| Prepuce | 1 | |
| Duct steno | 1 | |
| Withers | 5 | 7 |
| Foreign bodies. | | |
| Bone in throat | 1 | 1 |
| " foot | | 1 |
| " ear | | 1 |
| Forging | 1 | |
| Fractures. | | |
| Calcis | 1 | |
| Femur | 3 | |
| Humerus | 2 | 12 |
| Maxilla | 1 | |
| Metatarsal | 1 | |

| | HOSPITAL. | OUT PATIENTS. |
|---|---|---|
| Fractures (continued). | | |
| Pelvis | 4 | |
| Phalanges | 2 | |
| Radius | 2 | 1 |
| Ribs | 1 | |
| Tarsus | 1 | |
| Temporal bone | 1 | |
| Tibia | 4 | |
| Ulna | 1 | |
| Gastritis | 3 | 5 |
| " chronic | | 1 |
| Glanders* | 7 | 12 |
| Glaucoma | | 2 |
| Gravel | | 2 |
| Grease | 3 | 12 |
| Hæmorrhoids | 3 | |
| Hæmaturia | 1 | |
| Hæmatocele | 1 | |
| Hæmaglobinuria | 14 | 1 |
| Heat stroke | 1 | |
| Helminthes. | | |
| Ascaridies | 3 | 6 |
| Taeniæ | 2 | 3 |
| Immobility | 3 | 2 |
| Impaction of Rectum | 1 | 4 |
| Indigestion. | | |
| Chronic | 3 | 3 |
| Intestinal | 7 | 11 |
| Influenza | 4 | 1 |
| Interfering | 1 | 2 |
| Keratitis | 1 | 12 |
| Keraphyllocele | 1 | |
| Keratoma | | 1 |
| Knuckling | 1 | |
| Laminitis, acute | 7 | 1 |
| " chronic | 2 | |
| Laryngitis | 1 | 1 |
| Leucocythaemia | | 1 |
| Lymphangitis | | 1 |
| Mange, sarcoptic | 20 | 31 |
| " dermatoded | 1 | |
| Mammitis | 1 | |
| Meningitis | 2 | |
| Metritis | 1 | 4 |
| Navicular disease | 1 | 6 |
| Necrosis | 1 | |
| Nephritis | 1 | |
| Œdema | 1 | |
| Onychia | 1 | |
| Ophthalmia, periodic | 1 | 3 |
| " traumatic | 1 | 4 |
| Osteo porosis | 2 | 4 |
| Paralysis | 2 | |
| " traumatic | | 1 |
| Pralysis penis | 2 | |
| Paronychia | 1 | |
| Parturition | | 1 |
| Pemphigus | | 1 |
| Penis, deformed | | 1 |
| Periostitis | 2 | 4 |
| " rheumatic | 1 | |
| Pericarditis | | 2 |
| Peritonitis | | 1 |
| Pharyngitis | 1 | |
| Plethora | 1 | |
| Pleurisy | 2 | |
| Pneumonia | 6 | 1 |
| " broncho | 3 | 5 |
| Poisoning. | | |
| Laurel leaves | 1 | |
| Lead | 2 | |
| Mercurial | | 1 |
| Poll Evil | 3 | 16 |
| Pregnancy | 2 | |
| Pricked foot | 5 | 9 |
| Quarter cracks | 2 | 3 |
| Quittor | 9 | 3 |
| Rickets | | 1 |
| Ringbone | 9 | 10 |
| Rheumatism | 11 | 5 |
| Roaring | 1 | 6 |
| Sarcocele | | 1 |
| Schirrous cord | 1 | 1 |
| Shoe boil | 3 | 9 |
| Shoeing | | 417 |
| Sidesbone | 2 | 3 |
| Spavin | 15 | 48 |
| Spayed bitches | 1 | 2 |
| Splints | 1 | 3 |
| Strain of fetlock | 1 | |
| " suspensory ligaments | | 1 |
| Synovitis. | | |
| Carpal sheath | | 3 |
| Great sesamoid sheath | | 2 |
| Tail straightened | 1 | 1 |
| Tetanus | 5 | |
| Teeth filed | | 21 |
| " irregular | | 4 |
| " removed | | 6 |
| Thoroughpin | | 1 |
| Thrombosis | | 3 |
| Thrush | 2 | 12 |
| Toe crack | 2 | 7 |
| Tuberculosis† | 2 | 1 |
| Tumors. | | |
| Carcinoma | 4 | 4 |
| Epithelioma | 2 | 10 |
| Fibroma | 6 | 14 |
| Lympoma | 1 | 2 |
| Myxoma | 2 | 3 |
| Sarcoma | 3 | |
| Sebaceous | | 3 |
| Tympanitis | | 1 |
| Ulcers | 2 | 2 |
| Uterus prolapsus | 2 | |
| Vaccinia | 1 | |
| Vertigo | | 1 |
| Wounds. | | |
| Bites | 2 | |
| Contused | 1 | |
| Incised | 4 | 1 |
| Lacerated | 10 | 13 |
| Surgical | 2 | |
| Total. | 426 | 1128 |
| Total | | 1584 |

A large number of cases were treated for more than one disease which are not enumerated.

*The nineteen cases of Glanders-Farcy represent but a third of those which were submitted for diagnosis and autopsy during the year, as they were immediately consigned to a special shed and were not registered in the office. On all of these a complete and thorough autopsy was held, generally preceeded by a clinical lecture on the disease.

†A large number of cases of Tuberculosis in cattle were employed in the dissecting room during the year, these also failed to be registered, but served for clinical demonstration and post-mortem examination.

Cases Offering Special Points of Interest.—Several of these have already been reported in the Journal; among them the case of Myxoma of Choride Plexus in the horse (Article on Etiology of Tumors), case of Chronic Glanders (July number) et. al.

Hysteria in Mare, No. 781.—Bay mare, about fourteen years, partly cob bred; used for light carriage work. She is ordinarily quiet, but for some months at her horsing period she becomes absolutely unmanageable. She kicks and bites in the stall, destroying all the wood work The entrance of any one or any sudden noise starts her up into a paroxysm of mixed rage and fear. The harness is put on with difficulty; when harnessed she may drive quietly for a time, but with any unusual sight, or with no apparent cause, she will have attacks similar to those in the stable. At the end of a few days the trouble disappears for a variable period of several weeks. Examination of uterus and ovaries showed no abnormal condition. Treatment with large doses (dr.iii. several times daily).commenced with the first symptom, allayed the trouble; but, when ommitted, they returned with old severity. The case was lost sight of after six months.

Paralysis Penis (Sympathetic), No. 823.—Black gelding, six years old, coach horse. Was purchased three months previously. Penis has been gradually dropping, until it hangs from the sheath most of the time. No cause can be discovered, except a tumor on the cord of the testicle—off side—the near cord is very long and fastened to the cicatrix. Cast and removed a fibroma the size of a lemon. Recovery.

Knuckling in Zebra, No. 872.—Young zebra has been in close confinement for some time, but is in good condition except a knuckling of posterior fetlocks, which has rapidly increased for several weeks, attended with contraction of the normally very narrow hind feet. Cast and shod with shoes having an anterior projection three inches in length. Eight small nails were used and feet were pried into their proper position by the animal's own weight. Regular exercise was given at first, and later a more roomy box with dirt floor. When the feet had grown to a normal shape the knuckling had disappeared.

Chorea Poeto-Myclitis In Dog, No. 930.—A finely-bred colly dog, one year old, has just recovered from distemper. In good condition of nourishment, but has had chorea, increasing in severity, for two weeks. A peculiar movement of the fore legs developed, making the animal walk as if going upstairs. This was followed by the following convulsive stage: On movement he falls into spasms of right hand exterior muscles of head and neck, and exterior muscles of back and body. These are so great, when an attempt is made to place the animal on its feet, as to cause him to turn a complete somersault backwards, followed by violent convulsions, which end in a paralytic stage, lasting for some time; these terminate in other spasms. The animal lies helpless on his back or side with the head and neck twisted almost under the shoulder. He is perfectly conscious, and evinces his feelings when spoken to by the movement and expression of his hopeless eyes. The tail would wag at a word at almost all times. Liquid food and stimulants were given with difficulty. The liquor (Hydrag. et Potas. Iod.), was given, at first in two minim doses, three times a day, and gradually increased to ten minims, when diarrhœa developed. The Donovan's solution was then reduced and then again gradually increased. After a week of the apparently-hopeless stage, a slow improvement commenced and contiuued regularly until the animal made a perfect recovery, except for a slight twist of the head and a faint irregularity of movement—string halty—of the fore legs.

No 946. Collie bitch, 6 mo., same owner as last case. Apparent recovery from distemper followed in ten days by chorea, and later by complete paralysis of hind legs. The feet below the tarsus were in flexion so that the bitch in dragging herself around wore the anterior part of the phalanges bare. Same treatment as last case, in smaller doses, with friction and manipulation of muscles of hind quarter, followed by recovery in three months.*

CHRONIC METRITIS IN MARE. No. 987. Bay mare, fifteen years, rather well bred. Had foals at eight and nine years of age, again at eleven years, and since then has been horsing constantly. Has been served time and again but without success. For two years had been emitting a watery discharge trom vulver, in quantity and frequently. The effort to discharge usually accompanied by a severe straining. This had increased until the mare has become an offensive animal to drive. On manual examination in rectum and vagina, and examination of bladder by a sound, the latter organ proved normal. The os uterus was enlarged, flabby to the touch and congested. Excessive mucous from its surface was mixed with purulent matter. Two fingers passed easily into the uterus and allowed the escape of fluid. The internal surface was velvety to the touch. A rubber tube passed into the uterus drained it of some six quarts of a very fluid muco-pus. The case was treated by daily syphoning out the accumulation of fluid and injection of hot water 95° F., and Boracic acid. At first, ten quarts of water could be run in with a funnel and tube and then syphoned out. In a week's time it was reduced to four quarts, and the morning accumulation of muco pus was scarcely a quart. In four weeks the discharge was barely appreciable and the uterus would hold less than a quart of the injection. Treatment was ordered to be continued by the owner twice a week.

STRANGULATED HAEMORRHOIDS. No. 1129. Bay filly, yearling, found in pasture four days ago with a protrusion of mucous membrane in a circle from anus, which has been manipulated and rubbed with linaments before being brought for treatment. Find the part congested, œdematous and fixed in its position by distended veins in which are hard blood clots which, after an incision, can be shot from their place like peas from a pod. The distention completely blocks the rectum from which there is no evacuation in the last twenty-four hours. After relieving the congestion by puncture, enucleation of a number of the clots and evacuation of the rectum, find in forty-eight hours that the swelling cannot be reduced. Remove by ligatures of ordinary elastic letter bands stretched in and out from border of anus to healthy internal mucous membrane, in continuous figure of eight sutures. The part sloughed off in two days leaving a healthy wound which cicatrized in ten days.

R. S. HUIDEKOPER, Veterinarian.

*I have had a number of good results in other cases in the treatment of the sequelæ of distemper in dogs with Donovan's Solution and have obtained much better results than I have with Fowler's Solution or with any other form of treatment.

